# Species identification of introduced veronicellid slugs in Japan

**DOI:** 10.7717/peerj.13197

**Published:** 2022-04-22

**Authors:** Takahiro Hirano, Osamu Kagawa, Masanori Fujimoto, Takumi Saito, Shota Uchida, Daishi Yamazaki, Shun Ito, Shovon Mohammad Shariar, Takuo Sawahata, Satoshi Chiba

**Affiliations:** 1 Center for Northeast Asian Studies, Tohoku University, Sendai, Miyagi, Japan; 2 Graduate School of Life Sciences, Tohoku University, Sendai, Miyagi, Japan; 3 Faculty of Agriculture, Kindai University, Nara, Nara, Japan; 4 Department of Biology, Faculty of Science, Toho University, Funabashi, Chiba, Japan; 5 Department of Biochemistry and Molecular Biology, Rajshahi University, Rajshahi, Bangladesh

**Keywords:** COI, Mollusks, Veronicellidae, *Sarasinula plebeia*, *Semperula wallacei*, *Laevicaulis alte*, Greenhouses, Ryukyu Islands

## Abstract

Reliable identification of species is important for protecting native ecosystems against the invasion of non-native species. DNA barcoding using molecular markers, such as the mitochondrial cytochrome oxidase subunit 1 (COI) gene, helps researchers distinguish species. In this study, we focused on introduced veronicellid slugs in the Ryukyu Islands and some greenhouses on mainland Japan. Some veronicellids are medium-to-high risk pest species for humans. Identifying veronicellid species by their external morphology is difficult and unreliable because there is substantial overlap between intraspecific variation and interspecific differentiation. Therefore, internal morphologies such as male genitalia have been the primary traits used to distinguish veronicellids. To identify introduced veronicellid slugs in Japan to the species level, we used morphological assessment of male genitalia and DNA barcoding of the standard COI gene fragment. We also conducted species-delimitation analyses based on the genetic data. The results showed that five evolutionarily significant units, corresponding to four nominal species inhabit the Ryukyu Islands, of which two species were also found in the greenhouses of mainland Japan, including the first record of *Sarasinula plebeia* in Japan. The presence of non-native slug species could increase the transmission of parasites in Japan.

## Introduction

Introduced species often cause damage to native ecosystems and a decline in biological variation (Fritts & Rodda, 1998; Simberloff, Parker & Windle, 2005; Wetterer et al., 2006). Introduced species may also affect human health (Pimentel, Zuniga & Morrison, 2005). Parasite-infected hosts may transmit their parasites to other hosts (Lymbery et al., 2014). Moreover, introduced species can damage crops (Dean et al., 2016). The role of nursery trade in both the deliberate and accidental introduction of alien animal species is well known (White, Kramer & Hudler, 2010; Bergey et al., 2014). Similarly, botanical gardens, which function as places for the conservation of rare species and academic exhibitions, have been implicated in the early cultivation and/or introduction of various alien plants (Dawson et al., 2008; Hulme, 2011; Richling & von Proschwitz, 2021) and may also play an important role in introduction of animals.

In order to address the potential risks of introduced species it is in the first place important to be able to identify these alien species. Recent progress in molecular phylogenetics has contributed to resolving several taxonomic issues with respect to introduced species. DNA barcoding approach can provide important insights into interpreting the level of lineage diversity, particularly in taxonomically complex species group with similar external morphology. For instance, several studies have demonstrated that DNA barcoding can be a useful technique for studying invasive terrestrial slugs (Barr et al., 2009; Zemanova, Knop & Heckel, 2016; Zemanova et al., 2018; Dörler et al., 2018; Hutchinson et al., 2020).

Japan is a hotspot for terrestrial mollusks (*e.g*., Hirano et al., 2014, 2015; Cameron, 2016). In particular, the continental Ryukyu Islands and the oceanic Ogasawara Islands are small subtropical islands with unique geological backgrounds. Species diversity of terrestrial mollusks on these islands is high and includes several endemic lineages (*e.g*., Wada, Kameda & Chiba, 2013; Hirano, Kameda & Chiba, 2014; Hirano et al., 2018, 2019a; Chiba & Cowie, 2016). However, several species of terrestrial mollusks have been introduced to these islands, most likely by human activities such as plant nursery trade (*e.g*., Hirano et al., 2019d). In fact, approximately 100 species of terrestrial mollusks worldwide appear to be particularly well-adapted to environmental changes brought about by human activity, and often become highly abundant and characteristic components of invertebrate fauna in modified habitats (Barker, 2001; Cowie et al., 2008; Richling & von Proschwitz, 2021) and have the potential to invade new areas.

The family Veronicellidae is a group of terrestrial slugs with 23 recognized genera and 78 recognized species (Thomé, 1975) that are globally distributed throughout the tropics and subtropics (Gomes et al., 2010; Hirano et al., 2019d). In many countries and regions, these slugs are often found in human-related environments such as greenhouses and gardens (*e.g*., Brodie & Barker, 2012a; Ali & Robinson, 2020; Daglio et al., 2020). They feed on the leaves and stems of crops, defoliating and often killing plants (Rueda et al., 2002; Naranjo-García, Thomé & Castillejo, 2007; Constantino, Gomes & Benavides, 2010; Daglio et al., 2020). Some of the species are medium-to-high risk pests for humans. For example, they are intermediate hosts of *Angiostrongylus cantonensis* (Brodie & Barker, 2011; Kim et al., 2014), which causes angiostrongyliasis (Kim et al., 2014). Although Japan is not the native range of veronicellids, three species of veronicellid have been introduced to Japan and can be found in natural environments (Shimada, Makino & Hashiguchi, 1972; Hirano et al., 2019d): *Laevicaulis alte* (Férussac, 1822), *Semperula wallacei* (Issel, 1874), and Veronicellidae sp. have been recorded from the Ryukyu Islands and the Ogasawara Islands. In addition, *Laevicaulis alte* has been found in a greenhouse of Toyohashi Zoo and Botanical Park, Aichi Prefecture, temperate mainland Japan (Nishi & Matsuoka, 2009). Outside the greenhouse, there were also individuals that appeared to have been transferred along with discarded plants and waste materials from the greenhouse (Matsuoka, 2022, personal communication). However, this population may have not been established (Matsuoka, 2022, personal communication).

Although there is no native slug that can be confused with these veronicellids in Japan, identifying veronicellid species by their external morphology is difficult and unreliable because there is substantial overlap between intraspecific variation and interspecific differentiation (Cowie, 1997; Kim et al., 2016; Hirano et al., 2019d). Internal morphologies such as the shape of the penile complex, the penile gland, and an accessory structure to the penile complex have been the main ways to identify veronicoelids (Gomes & Thomé, 2004; Daglio et al., 2020). Nevertheless, among the three species introduced in Japan, *Lae. alte* can be distinguished from *Se. wallacei* and Veronicellidae sp. by its dark gray to nearly black body with a thin pale median dorsal line (Hirano et al., 2019d). In contrast, *Semperula wallacei* and Veronicellidae sp., which have brown bodies with gray spot, are very similar to each other (Hirano et al., 2019d). The genital morphology of these slug species including Veronicellidae sp. was not investigated in Hirano et al. (2019d). Moreover, identifying juveniles of these three species is challenging because juveniles of *Lae. alte* do not clearly show their taxonomic traits on their external morphology. Therefore, DNA barcoding is an important method for identifying these species. Since our previous work on DNA barcoding in Japanese veronicellids (Hirano et al., 2019d), we have continued to investigate the fauna of terrestrial mollusks on the main islands of the Ryukyu Islands. We collected additional samples of veronicellids from several islands of the Ryukyu Islands, as well as from greenhouses of multiple facilities in temperate mainland Japan. In this study, we identified the specimens based on DNA barcoding using COI sequences and male genital morphology. Here we report new records of *Se. wallacei* and Veronicellidae sp. on several islands. We also report *Lae. alte*, *Se. wallacei* and Veronicellidae sp. from new localities in Japan.

## Materials and Methods

### Samples

**Figure 1:**
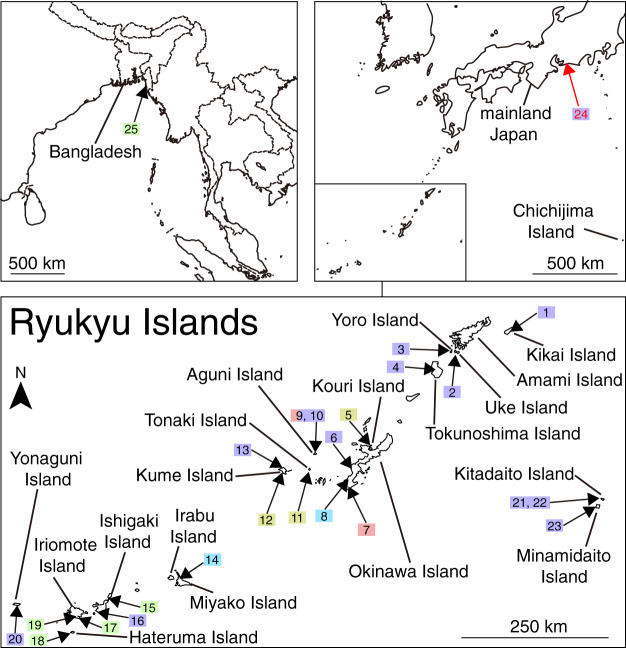
Map of the veronicellid sampling sites in Japan. Sites 1–23 are in the Ryukyu Islands, 24 is on mainland Japan, and 25 is in Bangladesh. The numbers correspond to the site number in Table S1. Toyohashi Zoo and Botanical Park (site 24) are indicated in red. Colours of squares reflect the sampling sites of the species as defined by the two species delimitation analyses (mlPTP and bPTP) in Fig. 2.

Sampling areas included both those known to harbour invasive species (public greenhouses) and natural environments. We collected 44 veronicellids from the Ryukyu Islands and five greenhouses from different facilities in temperate mainland Japan (Table S1; Fig. 1). Facilities other than Toyohashi Zoo and Botanical Park allowed us to collect and use samples, but did not agree to disclose their names and locations; and are therefore, identified as Greenhouses A–D (Table S1). We also collected an individual from Bangladesh. Sampling was conducted under the permission of Toyohashi Zoo and Botanical Park and Greenhouses A–D. In Japan, sampling outside of the greenhouses was conducted in areas where no permits were required. Sampling was also conducted under the support of Rajshahi University (Bangladesh) and this study complies with the Nagoya protocol. A tissue sample from each specimen was preserved in 99.5% ethanol for DNA extraction and the remaining soft bodies were stored in 70% ethanol for dissection. We deposited the specimens at Tohoku University Museum (Table S1). We used COI sequences from six individuals of three veronicellid species (*Lae. alte*, *Se. wallacei*, and Veronicellidae sp.) in Japan from our previous study (Hirano et al., 2019d), and obtained COI sequences of 181 individuals of other veronicellid species from GenBank and an individual from BOLD (Table S1). We also used COI sequences of two onchidiid individuals (*Onchidium vaigiense* and *Onchidella floridana*) from GenBank as outgroups (Hirano et al., 2019d).

**Figure 2:**
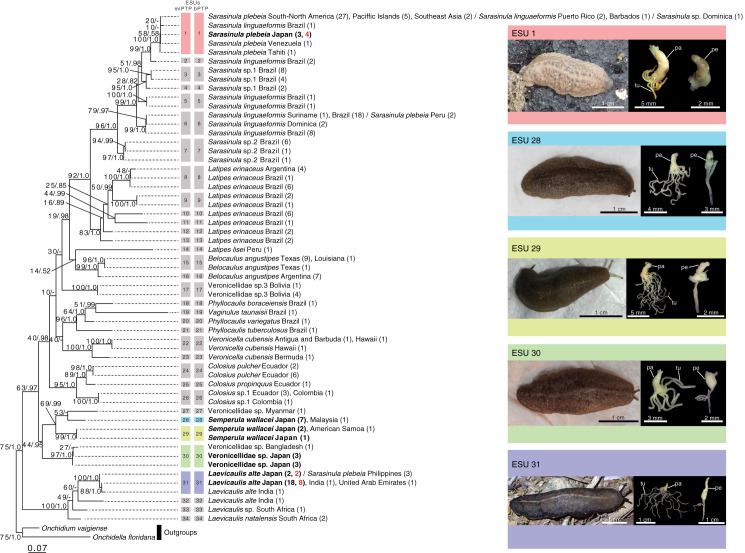
Maximum likelihood (ML) phylogenetic tree of the veronicellid slugs based on 470 bp of the COI gene showing the results of species delimitation analyses. Each tip label is a species name followed by the country/region name, and the number of individuals in brackets. Samples from Japan are indicated in bold. Samples from greenhouses are indicated in red. Vertical coloured bars to the right of the tree represent species as defined by the two species delimitation analyses (mlPTP and bPTP). Numbers on branches indicate ML bootstrap values followed by Bayesian posterior probabilities. Photographs of external morphology and male genitalia of the five species from Japan are shown. Each colour of the five species reflects the results of two species delimitation analyses. Digitiform gland and penis are left and right, respectively. pe, penis; pa, papilla of the digitiform gland; tu, tubules.

### COI sequence analyses

DNA was extracted from a fragment of the foot muscle of 48 slug individuals using a NucleoSpin Tissue kit (Macherey-Nagel, Düren, Germany) according to the manufacturer’s standard protocol. Polymerase chain reaction (PCR) conditions and primers for the amplification of the standard COI barcode are described by Hirano et al. (2019d). Sequencing was conducted by Eurofins Genomics (Ohta, Tokyo, Japan) using PCR products purified using Exo-SAP-IT (Amersham Biosciences, Little Chalfont, UK). Each sequence was deposited in the GenBank database (Table S1). The COI sequences were aligned with Muscle 3.8 (Edgar, 2004), based on unique sequences. We then performed phylogenetic analyses using maximum likelihood (ML) and Bayesian methods. We used Kakusan4-4.0.2011.05.28 (Tanabe, 2011) to select appropriate models of sequence evolution. For ML and Bayesian analyses, we used each AIC and BIC criteria, respectively [ML: GTR+G; Bayes: GTR+G+I (codon position 1), F81+I (codon position 2), and HKY+G (codon position 3)]. ML analysis was conducted using the selected models with RAxML HPC2 (Stamatakis, 2006). We assessed nodal support for ML analysis using bootstrap analyses with 1,000 replications. We conducted Bayesian analysis in MrBayes version 3.1.2 (Ronquist & Huelsenbeck, 2003) using two simultaneous runs, consisting of four simultaneous chains for 30 million generations and tree sampling every 1,000 generations. For all parameters, we ensured that the effective sample size (ESS) values were above 200. After log and tree files were checked by Tracer version 1.7 (Rambaut et al., 2018), we summarized the entire posterior distribution using the Monte Carlo Markov chain files in Treeannotator version 2.4.4 (BEAST package; burnin = 10%, maximum clade credibility, and mean heights). We conducted ML and Bayesian analyses through the CIPRES Science Gateway (Miller, Pfeiffer & Schwartz, 2010). Only posterior probabilities ≥0.95, were considered well-supported, and bootstrap values ≥75% were considered to reflect high support.

### Species delimitation analyses

To investigate species boundaries using molecular data, we conducted species-delimitation analyses using the web server at http://species.h-its.org/ptp/. ML Poisson Tree Process model (mlPTP; Zhang et al., 2013) and the Bayesian Poisson Tree Process model (bPTP; Zhang et al., 2013). In PTP, speciation or branching events are modeled in terms of number of mutations. For the mlPTP and bPTP analyses, the ML COI tree estimated above was used with default parameters (number of MCMC generation: 100,000; burn-in: 10%). For bPTP, we visually checked convergence of the likelihood plot of each delimitation.

### Morphological assessment of male genitalia

To investigate the interspecific variation in male genital traits such as penial morphology and digitiform gland, we examined genital anatomy of selected specimens representing the five evolutionarily significant units (ESUs, see the results) using a stereomicroscope. Adult individuals of the slugs were dissected (4 individuals of ESU 1, 6 individuals of ESU 28, 2 individuals of ESU 29, 4 individuals of ESU 30, and 9 individuals of ESU 31).

## Results

### Gene tree inference

The topologies of the trees from ML and Bayesian analyses were largely consistent, at least with regard to the relationships of the well-supported lineages (Fig. S1). The populations from the greenhouses included two major lineages. The specimens collected from Greenhouse D, Okinawa Island, and Aguni Island belonged to the genus *Sarasinula*. In particular, *Sa. plebeia*, *Sa. linguaeformis*, and *Sarasinula* sp. were closely related to the Japanese specimens. Specimens from Toyohashi Zoo and Botanical Park, Greenhouses A–D, Uke Island, Yoro Island, Amami Island, Tokunoshima Island, Kikai Island, Okinawa Island, Aguni Island, Kume Island, Ishigaki Island, Yonaguni Island, Kitadaito Island, and Minamidaito Island were closely related to GenBank sequences assigned to *Lae. alte* from India and United Arab Emirates, and *Sa. plebeia* from Philippines. Specimens of Veronicellidae sp. from Japan clustered with the individual from Bangladesh. In the Bayesian tree, specimens from Irabu Island, Miyako Island, Okinawa Island, Kouri Island, Tonaki Island, and Kume Island formed a clade with GenBank sequences assigned to *Se. wallacei* from Malaysia and American Samoa. Veronicellidae sp. from Myanmar was sister to this clade. The ML tree, differed from the Bayesian tree in that the populations from Miyako Island, Okinawa Island, and Chichijima Island, and *Se. wallacei* from Malaysia were not monophyletic with the populations from Kouri Island, Kume Island, and Tonaki Island, and *Se. wallacei* from American Samoa.

### Species delimitation

The two species-delimitation tests both estimated the same number of species (34 ESUs) (Fig. 2; Table S1). These ESUs were well-supported by bootstrap values and posterior probabilities (Fig. 2; Fig. S1). Three ESUs (ESU 1, ESU 6, and ESU 31) have specimens attributed to multiple species, but other ESUs only included specimens assigned to a single species. The individuals collected in this study belonged to four ESUs (ESU 1: *Sa. plebeia* + *Sa. linguaeformis* + *Sarasinula* sp., ESU 28: *Se. wallacei* A, ESU 29: *Se. wallacei* B, ESU 30: Veronicellidae sp., and ESU 31: *Lae. alte* + *Sa. plebeia*). The populations from the greenhouses included two ESUs (ESU 1: *Sa. plebeia* + *Sa. linguaeformis* + *Sarasinula* sp. and ESU 31: *Lae. alte* + *Sa. plebeia*). *Sarasinula plebeia*, *Sa. linguaeformis*, *Latipes erinaceus*, *Belocaulus angustipes*, *Veronicella cubensis*, *Se. wallacei*, and *Lae. alte* were split into multiple ESUs.

### Genital morphology

The individuals collected in this study belonged to five ESUs showed the following morphological traits of male genitalia (Fig. 2; Table 1; Table S2); ESU 1: penis short, smooth, with a small glans. Digitiform gland with a papilla with 4–8 short tubules with varying lengths (not bifurcate). ESUs 28: penis short, smooth, with a small glans. Digitiform gland with a papilla with 12–16 long tubules with varying lengths (bifurcate). ESUs 29: penis short, smooth, with a small glans. Digitiform gland with a papilla with 11–16 long tubules with varying lengths (bifurcate). ESU 30: penis short, smooth, with a small glans. Digitiform gland with a papilla with 5–9 short tubules with varying lengths (not bifurcate). ESU 31: penis long, smooth, with a long glans. Digitiform gland with a papilla with 10–18 long tubules with varying lengths (bifurcate). There was no significant variation within each ESU in the external shape of the penis.

**Table 1:** The morphological results for the five ESUs, native range and non-native areas of the slugs, and references to the first time the slugs have been noted in Japan.

ESU (Fig. 2)	Taxa	External morphology	Genital morphology		Native area	Non-native area	References
			Penis	Digitiform gland			
ESU 1	*Sarasinula plebeia*	Light to dark mottled brown body with dark spot	Short, smooth, with a small glans	With a papilla with 4–8 short tubules with varying lengths (not bifurcate)	South America	Japan, North and Central America, West Indies, Asia, Africa, Australia, Fiji, Hawaii, Indonesia, Marianas, New Caledonia, Philippines, Solomon Islands, Tahiti, Tuamotu, Vanuatu, Western Samoa, West Islands	This study
ESU 28	*Semperula wallacei*	Yellow to brown body with gray spot	Short, smooth, with a small glans	With a papilla with 12–16 long tubules with varying lengths (bifurcate)	Southeast Asia	Japan, Christmas Island, China, Fiji, Indonesia, Western Samoa, Vanuatu, Virgin Islands, Guadeloupe, Martinique, Grenada, Barbados	Hirano et al. (2019d)
ESU 29	*Semperula wallacei*	Yellow to brown body with gray spot	Short, smooth, with a small glans	With a papilla with 11–16 long tubules with varying lengths (bifurcate)	Southeast Asia	Japan, Christmas Island, China, Fiji, Indonesia, Western Samoa, Vanuatu, Virgin Islands, Guadeloupe, Martinique, Grenada, Barbados	Hirano et al. (2019d)
ESU 30	Veronicellidae sp.	Brown body with gray spot	Short, smooth, with a small glans	With a papilla with 5–9 short tubules with varying lengths (not bifurcate)	South Asia?	Japan	Hirano et al. (2019d)
ESU 31	*Laevicaulis alte*	Dark gray to nearly black body with a thin pale median dorsal line	Long, smooth, with a long glans	With a papilla with 10–18 long tubules with varying lengths (bifurcate)	Africa	Japan, India, Sri Lanka, Taiwan, Hong Kong, Qatar, Saudi Arabia, northern Australia, Hawaii, New Caledonia, Vanuatu, Samoa, American Samoa, Bermudas, Texas, Indonesia, Malaysia, Philippines, New Guinea, New Caledonia, Fiji, Egypt	Shimada, Makino & Hashiguchi (1972)

**Note:**

“Non-native area” of *Se. wallacei* might include some natives, and it is not easy to identify the species or the range. For convenience, therefore, we have listed all areas where the species was recorded, as “non-native area”.

## Discussion

Our results clarified the species diversity of introduced veronicellid slugs in Japan by using a combined approach of both DNA barcoding and anatomical studies of genitalia (Fig. 2; Table 1). Our studies identified five veronicellid ESUs corresponding to four nominal species living in Japan: *Sa. plebia* (ESU 1), Veronicellidae sp. (ESU 30), *Lae. alte* (ESU 31), *Se. wallacei* (ESU 28 and ESU 29) (Fig. S1; Fig. 2). ESU 1 includes specimens identified in this study as *Sa. plebia* from multiple localities, along with from GenBank attributed to *Sarasinula* sp. and *Sa. lingauaeformis*. The latter specimen may have been misidentified in previous studies. Alternatively, as our results are based on a single mitochondrial gene, these results could accurately reflect incomplete lineage sorting or/and hybridization between species (*i.e*., Hirano et al., 2019b, 2019c). Conversely, ESU 28 and ESU 29 both include specimens collected from multiple localities, and all specimens in these clades are currently recognised as *Se. wallacei*, raising the possibility of cryptic species. From the above, it is difficult to evaluate the introduced species diversity based on DNA barcoding alone. The present study thus emphasizes the necessity to integrate molecular data with assessments of traits that reflect species differences to properly interpret the species delimitation.

Considering disagreements between morphospecies registered in the sequence database and the estimated ESUs, anatomical morphology can help us to evaluate the species diversity with the species delimitation results (Lukic et al., 2021). Five ESUs in our sites can be largely distinguished into four morphological patterns (Fig. 2; Table 1). ESU 1 included sequences assigned to three species: *Sa. plebeia*, *Sarasinula* sp., and *Sa. linguaeformis* (Fig. 2). Genitalia of *Sa. plebeia* differs from that of *Sa. linguaeformis* by the number of tubules on the digitiform gland: *Sa. plebeia* has 4–8, whereas *Sa. linguaeformis* has 8–19 (Gomes, 2009; Oliveira Rocha, 2019). The morphological traits of the individuals we collected belonging to ESU 1 are consistent with that of *Sa. plebeia*. Although morphological data for *Sarasinula* sp. in ESU 1 are lacking, the individuals of ESU 1 from Japan can be treated as *Sa. plebeia*. *Laevicaulis alte* and *Sa. plebeia* composed ESU 31 (Fig. 2). The individuals we collected belonging to ESU 31 can be treated as *Lae. alte* based on the consistency of the external morphology such as black body with a thin pale median dorsal line, and male genitalia such as elongated penis (Kim et al., 2016). ESU 28 was morphologically similar to ESU 29 (Fig. 2; Table 1), and these two ESUs were well-supported as a monophyletic lineage (Bayesian posterior probability = 0.99; Fig. 2; Fig. S1). These ESUs can be identified as *Se. wallacei* based on the consistency of the external morphology such as yellow to brown body and male genitalia such as shape of penis and number of tubules of digitiform gland (16) (Forcart, 1969). We found no morphological traits that could distinguish ESU 28 and ESU 29 and they may be cryptic species, because the genetic distance between them is approximately 12%. If there are cryptic species in *Se. wallacei*, a clear idea of the geographic distribution of each relies on using specimens for which there are sequence data. In fact, cryptic species may be common in Veronicellidae, with multiple ESUs found in *Sa. linguaeformis* (ESU 1, ESU 2, ESU 5 and ESU 6), *Lat. erinaceus* (ESU 8–13), *B. angustipes* (ESU 15 and ESU 16), *V. cubensis* (ESU 22 and ESU 23) (Fig. 2). In this study, we conservatively treat ESU 28 and ESU 29 as a single species because there are no morphological differences in genitalia or external appearance, and some species delimitation methods are known to oversplit (Lukic et al., 2021). ESU 30 has also very similar external morphology to that of ESU 28 and ESU 29, but pattern of genitalia such as the digitiform gland was clearly different (Fig. 2; Table 1). Therefore, we identified four nominal veronicellid species (*Lae. alte*, *Sa. plebeia*, *Se. wallacei* and Veronicellidae sp.) in our sites, with *Sa. plebeia*, being a new record for Japan (Table 1). *Laevicaulis alte* and *Sa. plebeia* were found in all greenhouses and greenhouse D, respectively (Fig. 1; Table S1). These species were also found in the Ryukyu Islands (Fig. 1; Table S1). *Semperula wallacei* and Veronicellidae sp. were only found in pristine areas far from greenhouses (Fig. 1; Table S1).

Veronicellidae sp. from Japan and Bangladesh is an undescribed species or genetic data in GenBank/BOLD requires investigation by taxonomic experts. The slugs introduced to Japan might have originated from four different sources (Table 1): South Asia (Veronicellidae sp.), Africa (*Lae. alte*: Brodie & Barker, 2012a), Southeast Asia (*Se. wallacei*: Gomes & Thomé, 2004; Gomes et al., 2010), and South America (*Sa. plebeia*: Brodie & Barker, 2012b; Daglio et al., 2020). However, it is also possible that Japanese populations are not derived from these species’ native regions, and they may have been sourced from other areas they have been introduced (see Table 1 for non-native ranges). *Semperula wallacei* has been recorded from Christmas Island, China, Fiji, Indonesia, Western Samoa, Vanuatu, Virgin Islands, Guadeloupe, Martinique, Grenada, and Barbados (Gomes & Thomé, 2004; Molet, 2014), but some of these distribution areas may be non-native areas (Table 1). The presence of non-native slug species may increase the transmission of parasites in Japan and raises the potential for new crop pests.

For introduced veronicellids, the potential for colonization success and expansion of distribution may depend on temperature (Lanza & Quattrini, 1964; Raut & Panigrahi, 1988; Sommer & Cowie, 2020), and temperature differences might indirectly affect the potential for introduction in terms of differences in food. A small number of veronicellid individuals can quickly proliferate, facilitating rapid colonization of a new location (Sommer & Cowie, 2020). *Laevicaulis alte* can mate and lay eggs within a day, and a single *Lae. alte* individual maintained alone from hatching laid fertilized eggs (Lanza & Quattrini, 1964; Sommer & Cowie, 2020). Therefore, veronicellid slugs can easily establish populations within a suitable habitat and temperature. A previous study on growth, reproduction, and their relationship to temperature in *V. cubensis* and *Lae. alte* showed that, for both species, the time taken for eggs to hatch was less at 27 °C than at 22 °C (Sommer & Cowie, 2020). Eggs of *Lae. alte* and *Sa. plebeia* failed to hatch at 10 °C, 15 °C, and 35 °C, and 15 °C, respectively (Raut & Panigrahi, 1988; Rueda Pinzon, 1989). Although the average temperature in Japan has risen at a rate of 1.24 °C in the past 100 years, the minimum temperature of the mainland is generally less than 0 °C in winter (Japan Meteorological Agency, 2021). For example, the average temperature of mainland Japan (Tokyo) was 15.4 °C from 1981 to 2010, while that of Okinawa Island (Naha) was 23.1 °C (Japan Meteorological Agency, 2021). Veronicellids are tropical and subtropical species, so they must adapt to temperature in order to establish populations outside their native habitats. Although it is possible introduced veronicellids will expand their distribution within the Ryukyu Islands, establishing populations in mainland Japan may be more difficult. This is consistent with the extinction of the population outside the greenhouse in Toyohashi Zoo and Botanical Park (Matsuoka, personal communication). Greenhouses are maintained at warmer temperatures, and so this may explain how they can survive in greenhouses on the mainland. Opportunities for migration may, however, create opportunities for evolutionary change, such as resistance to different temperature zones (Park et al., 2012), so it is necessary to pay attention to introduced veronicellids even if the slugs are presently not adapted to non-native temperatures.

Even though it has been only a few years since the previous study (Hirano et al., 2019d), the present study demonstrates the invaded area of veronicellid slugs in Japan has expanded and records the presence of a new species. Future detailed morphological studies and genome-wide genetic analyses are needed to disentangle relationships between ESU 28 and ESU 29, and it is possible that the number of species might increase. In order to prevent the translocation of these invasive species, the importance of domestic plant quarantines has also been highlighted in addition to national plant quarantines (Bergey et al., 2014). Mutual quarantine between the shipping and receiving sites would help prevent the introduction of non-native organisms. Continuing surveys of terrestrial malaco-fauna are needed to clarify how frequently molluskan species invade and establish populations in non-native regions.

## Conclusion

Our combined approach based on DNA barcoding and genital anatomy clarified species diversity of introduced veronicellid species in Japan. We identified five molecularly delimited ESUs, corresponding to four nominal veronicellid species (*Lae. alte*, *Sa. plebeia*, *Se. wallacei* and Veronicellidae sp.) in our sites, with *Sa. plebeia*, being a new record for Japan. Two of the species (*Lae. alte* and *Sa. plebeia*) were also found in the greenhouses of mainland Japan. The presence of non-native slug species is of concern as it could increase the transmission of parasites in Japan.

## Supplemental Information

10.7717/peerj.13197/supp-1 Supplemental Information 1 Phylogenetic trees of the veronicellid slugs based on 470 bp of the COI gene. (A) Maximum likelihood (ML) tree; (B) Bayesian tree. Each tip label is a species name followed by the locality, and the GenBank accession number in brackets. Samples from Japan are indicated in bold. Samples from greenhouses are indicated in red. Numbers on branches indicate ML bootstrap values and Bayesian posterior probabilities, respectively.

10.7717/peerj.13197/supp-2 Supplemental Information 2 Sampling sites, ESU, GenBank or BOLD accession number of each DNA sequences, and specimen ID of each sample in Tohoku University.

10.7717/peerj.13197/supp-3 Supplemental Information 3 Variation of number of the tubes of the digitiform gland in each ESU.

10.7717/peerj.13197/supp-4 Supplemental Information 4 Sequences.

## Additional Information and Declarations

### DNA Deposition

The following information was supplied regarding the deposition of DNA sequences: New sequences are available at GenBank: LC636077–LC636119 and LC636218–LC636222. It is not publicly available yet, so we uploaded the sequence as a Supplemental File 1 (COI.fas).

### Data Availability

The following information was supplied regarding data availability: The sequences in the study are available in the Supplemental File and at GenBank: LC636077–LC636119 and LC636218–LC636222.
