# Supplementary figures and images for: Species identification of introduced veronicellid slugs in Japan
Source: PeerJ. 2022 Apr 22;10:e13197. doi: 10.7717/peerj.13197 (PMC9037128; doi:10.7717/peerj.13197)

A:ML

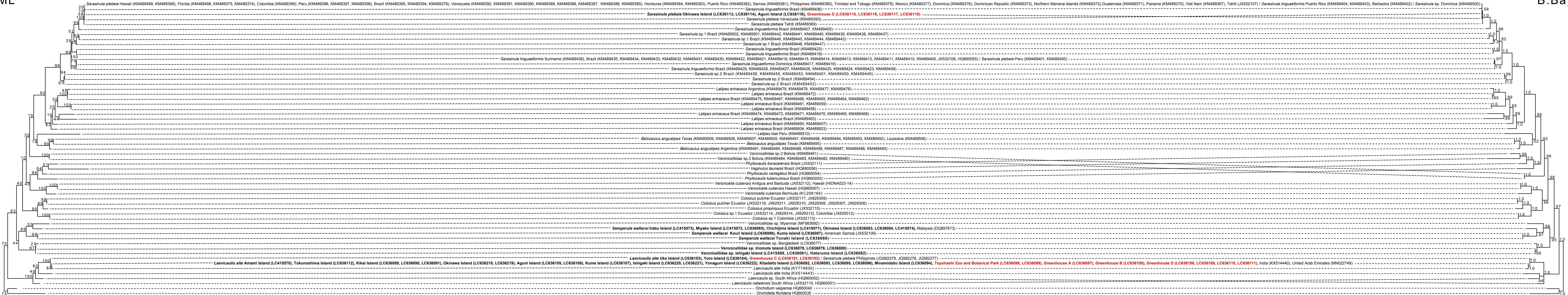

B:Bayes

Supplement: Supplemental Information 1 — (A) Maximum likelihood (ML) tree; (B) Bayesian tree. Each tip label is a species name followed by the locality, and the GenBank accession number in brackets. Samples from Japan are indicated in bold. Samples from greenhouses are indicated in red. Numbers on branches indicate ML bootstrap values and Bayesian posterior probabilities, respectively. [file peerj-10-13197-s001.pdf]
