# Supplementary material for: Species identification of introduced veronicellid slugs in Japan
Source: PeerJ. 2022 Apr 22;10:e13197. doi: 10.7717/peerj.13197 (PMC9037128; doi:10.7717/peerj.13197)
Supplement: Supplemental Information 2 [file peerj-10-13197-s002.docx]

**Table S1.** Sampling sites, ESU, GenBank or BOLD accession number of each DNA sequences, and specimen ID of each sample in Tohoku University.

| Sampling site | | Accession number | ESU (Fig. 2) | Specimen ID in Tohoku University |
| --- | --- | --- | --- | --- |
| No. | Name |  |  |  |
| **Samples collected by authors** | |  |  |  |
| ***Laevicaulis alte*** | |  |  |  |
| 1 | Kikai Island, Kagoshima, Japan | LC636089 | ESU 31 | TUMo-200316-5 |
| 1 | Kikai Island, Kagoshima, Japan | LC636090 | ESU 31 | TUMo-HC4057 |
| 1 | Kikai Island, Kagoshima, Japan | LC636091 | ESU 31 | TUMo-HC4058 |
| 2 | Uke Island, Kagoshima, Japan | LC636103 | ESU 31 | TUMo-u516 |
| 3 | Yoro Island, Kagoshima, Japan | LC636104 | ESU 31 | TUMo-yoro |
| 4 | Tokunoshima Island, Kagoshima, Japan | LC636112 | ESU 31 | TUMo-YC2431 |
| 6 | Kadena, Okinawa Island, Okinawa, Japan | LC636218 | ESU 31 | TUMo-kin17 |
| 6 | Kadena, Okinawa Island, Okinawa, Japan | LC636219 | ESU 31 | TUMo-kin18 |
| 9 | Aguni Island, Okinawa, Japan | LC636105 | ESU 31 | TUMo-A2020o48 |
| 10 | Aguni Island, Okinawa, Japan | LC636106 | ESU 31 | TUMo-C2020o42 |
| 13 | Kume Island, Okinawa, Japan | LC636107 | ESU 31 | TUMo-G2020o19 |
| 16 | Minaminuhama, Ishigaki Island, Okinawa, Japan | LC636220 | ESU 31 | TUMo-kin19 |
| 16 | Minaminuhama, Ishigaki Island, Okinawa, Japan | LC636221 | ESU 31 | TUMo-kin20 |
| 20 | Yonaguni Island, Okinawa, Japan | LC636222 | ESU 31 | TUMo-kin23 |
| 21 | Kitadaito Island, Okinawa, Japan | LC636092 | ESU 31 | TUMo-kin12 |
| 21 | Kitadaito Island, Okinawa, Japan | LC636093 | ESU 31 | TUMo-HC8630 |
| 22 | Kitadaito Island, Okinawa, Japan | LC636095 | ESU 31 | TUMo-HC8892 |
| 22 | Kitadaito Island, Okinawa, Japan | LC636096 | ESU 31 | TUMo-HC8893 |
| 23 | Minamidaito Island, Okinawa, Japan | LC636094 | ESU 31 | TUMo-HC8657 |
| 24 | Toyohashi Zoo and Botanical Park (Greenhouse), Toyohashi, Aichi, Japan | LC636098 | ESU 31 | TUMo-kin11 |
| 24 | Toyohashi Zoo and Botanical Park (Greenhouse), Toyohashi, Aichi, Japan | LC636099 | ESU 31 | TUMo-kin12 |
|  | Greenhouse A | LC636097 | ESU 31 | TUMo-kin10 |
|  | Greenhouse B | LC636100 | ESU 31 | TUMo-kin13 |
|  | Greenhouse C | LC636101 | ESU 31 | TUMo-kin15 |
|  | Greenhouse C | LC636102 | ESU 31 | TUMo-kin16 |
|  | Greenhouse D | LC636108 | ESU 31 | TUMo-kin24 |
|  | Greenhouse D | LC636109 | ESU 31 | TUMo-kin25 |
|  | Greenhouse D | LC636110 | ESU 31 | TUMo-kin26 |
|  | Greenhouse D | LC636111 | ESU 31 | TUMo-kin27 |
| ***Salasinula plebeia*** | |  |  |  |
| 7 | Itoman, Okinawa Island, Okinawa, Japan | LC636113 | ESU 1 | TUMo-95 |
| 7 | Itoman, Okinawa Island, Okinawa, Japan | LC636114 | ESU 1 | TUMo-96 |
| 9 | Aguni Island, Okinawa, Japan | LC636118 | ESU 1 | TUMo-E2020o48 |
|  | Greenhouse D | LC636115 | ESU 1 | TUMo-kin6 |
|  | Greenhouse D | LC636116 | ESU 1 | TUMo-kin7 |
|  | Greenhouse D | LC636117 | ESU 1 | TUMo-kin28 |
|  | Greenhouse D | LC636119 | ESU 1 | TUMo-kin30 |
| ***Semperula wallacei*** | |  |  |  |
| 5 | Kouri Island, Okinawa, Japan | LC636086 | ESU 29 | TUMo-HC12299 |
| 8 | Naha, Okinawa Island, Okinawa, Japan | LC636083 | ESU 30 | TUMo-kin8 |
| 8 | Naha, Okinawa Island, Okinawa, Japan | LC636084 | ESU 30 | TUMo-kin9 |
| 11 | Tonaki Island, Okinawa, Japan | LC636088 | ESU 29 | TUMo-F2020o33 |
| 12 | Kume Island, Okinawa, Japan | LC636087 | ESU 29 | TUMo-D2020o15 |
| 14 | Miyako Island, Okinawa, Japan | LC636085 | ESU 30 | TUMo-HC6219 |
| **Veronicellidae sp. *sense* Hirano et al. (2019d)** | |  |  |  |
| 15 | Nosoko, Ishigaki Island, Okinawa, Japan | LC636081 | ESU 30 | TUMo-kin5 |
| 17 | Toyohara, Iriomote Island, Okinawa, Japan | LC636078 | ESU 30 | TUMo-kin1 |
| 17 | Toyohara, Iriomote Island, Okinawa, Japan | LC636080 | ESU 30 | TUMo-kin4 |
| 18 | Hateruma Island, Okinawa, Japan | LC636082 | ESU 30 | TUMo-kin22 |
| 19 | Haemi, Iriomote Island, Okinawa, Japan | LC636079 | ESU 30 | TUMo-kin3 |
| 25 | Cox’s Bazar, Bangladesh | LC636077 | ESU 30 | TUMo-HC9751 |
| **DNA database (GenBank and BOLD)** | |  |  |  |
| ***Belocaulus angustipes*** | |  |  |  |
|  | McAllen, Hidalgo, Texas, USA | KM489509 | ESU 15 |  |
|  | McAllen, Hidalgo, Texas, USA | KM489508 | ESU 15 |  |
|  | McAllen, Hidalgo, Texas, USA | KM489507 | ESU 15 |  |
|  | Harvey, Louisiana, USA | KM489506 | ESU 15 |  |
|  | McAllen, Hidalgo, Texas, USA | KM489505 | ESU 15 |  |
|  | McAllen, Hidalgo, Texas, USA | KM489497 | ESU 15 |  |
|  | McAllen, Hidalgo, Texas, USA | KM489496 | ESU 15 |  |
|  | McAllen, Hidalgo, Texas, USA | KM489494 | ESU 15 |  |
|  | McAllen, Hidalgo, Texas, USA | KM489493 | ESU 15 |  |
|  | McAllen, Hidalgo, Texas, USA | KM489492 | ESU 15 |  |
|  | McAllen, Hidalgo, Texas, USA | KM489495 | ESU 15 |  |
|  | San Miguel de Tucuman, Tucuman, Argentina | KM489491 | ESU 16 |  |
|  | Horco Molle, Yerba Buena, Tucuman, Argentina | KM489490 | ESU 16 |  |
|  | Horco Molle, Yerba Buena, Tucuman, Argentina | KM489489 | ESU 16 |  |
|  | Horco Molle, Yerba Buena, Tucuman, Argentina | KM489488 | ESU 16 |  |
|  | Horco Molle, Yerba Buena, Tucuman, Argentina | KM489487 | ESU 16 |  |
|  | Horco Molle, Yerba Buena, Tucuman, Argentina | KM489486 | ESU 16 |  |
|  | Horco Molle, Yerba Buena, Tucuman, Argentina | KM489485 | ESU 16 |  |
| ***Colosius propinquus*** | |  |  |  |
|  | Imbabura, Ecuador | JX532115 | ESU 25 |  |
| ***Colosius pulcher*** | |  |  |  |
|  | Pichincha, Ecuador | JX532116 | ESU 24 |  |
|  | Napo, Ecuador | JX532117 | ESU 24 |  |
|  | Provincia de Napo, Ecuador | JX629309 | ESU 24 |  |
|  | Provincia Pichincha, Ecuador | JX629311 | ESU 24 |  |
|  | Provincia Pichincha, Ecuador | JX629310 | ESU 24 |  |
|  | Provincia Pichincha, Ecuador | JX629308 | ESU 24 |  |
|  | Provincia Pichincha, Ecuador | JX629307 | ESU 24 |  |
|  | Provincia Pichincha, Ecuador | JX629306 | ESU 24 |  |
| ***Colosius* sp.1 *sense* Gomes et al. (2013)** | |  |  |  |
|  | Colombia | JX532113 | ESU 26 |  |
|  | Napo, Ecuador | JX532114 | ESU 26 |  |
|  | Provincia Pichincha, Ecuador | JX629314 | ESU 26 |  |
|  | Neira, Caldas, Colombia | JX629313 | ESU 26 |  |
|  | Colombia | JX629312 | ESU 26 |  |
| ***Laevicaulis alte*** | |  |  |  |
|  | Amami Island, Kagoshima, Japan | LC415570 | ESU 31 | TUMo-HC2245 |
|  | India | KY774830 | ESU 31 |  |
|  | Selapadu, Guntur-District, Andhra Pradesh, India | KX514440 | ESU 31 |  |
|  | Selapadu, Guntur-District, Andhra Pradesh, India | KX514443 | ESU 32 |  |
|  | Dubai, United Arab Emirates | MN022749 | ESU 31 |  |
| ***Laevicaulis natalensis*** | |  |  |  |
|  | Natal, South Africa | JX532110 | ESU 34 |  |
|  | South Africa | HQ660051 | ESU 34 |  |
| ***Laevicaulis* sp. *sense* Dayrat et al. (2011)** | |  |  |  |
|  | South Africa | HQ660052 | ESU 33 |  |
| ***Latipes erinaceus*** | |  |  |  |
|  | Tucuman, Tucuman, Argentina | KM489479 | ESU 8 |  |
|  | Horco Molle, Yerba Buena, Tucuman, Argentina | KM489478 | ESU 8 |  |
|  | Horco Molle, Yerba Buena, Tucuman, Argentina | KM489477 | ESU 8 |  |
|  | Horco Molle, Yerba Buena, Tucuman, Argentina | KM489476 | ESU 8 |  |
|  | Ernestina, Rio Grande do Sul, Brazil | KM489472 | ESU 8 |  |
|  | Florianopolis, Santa Catarina, Brazil | KM489475 | ESU 8 |  |
|  | Santa Cruz do Sul, Rio Grande do Sul, Brazil | KM489467 | ESU 8 |  |
|  | Santa Cruz do Sul, Rio Grande do Sul, Brazil | KM489466 | ESU 8 |  |
|  | Santa Cruz do Sul, Rio Grande do Sul, Brazil | KM489465 | ESU 8 |  |
|  | Cachoeirinha, Rio Grande do Sul, Brazil | KM489464 | ESU 8 |  |
|  | Sananduva, Rio Grande do Sul, Brazil | KM489462 | ESU 8 |  |
|  | Sao Paulo, Sao Paulo, Brazil | KM489461 | ESU 9 |  |
|  | Sao Paulo, Sao Paulo, Brazil | KM489459 | ESU 9 |  |
|  | Sao Paulo, Sao Paulo, Brazil | KM489458 | ESU 9 |  |
|  | Pinhalzinho, Sao Paulo, Brazil | KM489474 | ESU 10 |  |
|  | Pinhalzinho, Sao Paulo, Brazil | KM489473 | ESU 10 |  |
|  | Ernestina, Rio Grande do Sul, Brazil | KM489471 | ESU 10 |  |
|  | Ernestina, Rio Grande do Sul, Brazil | KM489470 | ESU 10 |  |
|  | Ernestina, Rio Grande do Sul, Brazil | KM489469 | ESU 10 |  |
|  | Ernestina, Rio Grande do Sul, Brazil | KM489468 | ESU 10 |  |
|  | Pinhalzinho, Sao Paulo, Brazil | KM489463 | ESU 11 |  |
|  | Sao Paulo, Sao Paulo, Brazil | KM489460 | ESU 12 |  |
|  | Serra da Cantareira, Sao Paulo, Brazil | KM489457 | ESU 12 |  |
|  | Brazil | KM489504 | ESU 13 |  |
|  | Brazil | KM489503 | ESU 13 |  |
| ***Latipes lisei*** | |  |  |  |
|  | Peru | KM489510 | ESU 14 |  |
| ***Phyllocaulis boraceiensis*** | |  |  |  |
|  | Sao Paulo, Brazil | JX532111 | ESU 18 |  |
| ***Phyllocaulis tuberculosus*** | |  |  |  |
|  | Brazil | HQ660053 | ESU 21 |  |
| ***Phyllocaulis variegatus*** | |  |  |  |
|  | Brazil | HQ660054 | ESU 20 |  |
| ***Sarasinula linguaeformis*** | |  |  |  |
|  | Minas Gerais, Brazil | JX532108 | ESU 6 |  |
|  | Puerto Rico | KM489404 | ESU 1 |  |
|  | Puerto Rico | KM489403 | ESU 1 |  |
|  | Graeme Hall, Christchurch Parish, Barbados | KM489402 | ESU 1 |  |
|  | Eusebio, Ceara, Brazil | KM489406 | ESU 1 |  |
|  | Eusebio, Ceara, Brazil | KM489407 | ESU 2 |  |
|  | Eusebio, Ceara, Brazil | KM489408 | ESU 6 |  |
|  | Eusebio, Ceara, Brazil | KM489405 | ESU 2 |  |
|  | Cristalina, Goias, Brazil | KM489420 | ESU 5 |  |
|  | Cristalina, Goias, Brazil | KM489418 | ESU 5 |  |
|  | Saramacca, Saramacca, Suriname | KM489436 | ESU 6 |  |
|  | Campos dos Goytacazes, Rio de Janeiro, Brazil | KM489435 | ESU 6 |  |
|  | Campos dos Goytacazes, Rio de Janeiro, Brazil | KM489434 | ESU 6 |  |
|  | Campos dos Goytacazes, Rio de Janeiro, Brazil | KM489433 | ESU 6 |  |
|  | Campos dos Goytacazes, Rio de Janeiro, Brazil | KM489432 | ESU 6 |  |
|  | Campinas, Sao Paulo, Brazil | KM489431 | ESU 6 |  |
|  | Campinas, Sao Paulo, Brazil | KM489430 | ESU 6 |  |
|  | Cristalina, Goias, Brazil | KM489422 | ESU 6 |  |
|  | Cristalina, Goias, Brazil | KM489421 | ESU 6 |  |
|  | Cristalina, Goias, Brazil | KM489419 | ESU 6 |  |
|  | Brazil, Rio de Janeiro, Rio de Janeiro, Brazil | KM489415 | ESU 6 |  |
|  | Manaus, Amazonas, Brazil | KM489414 | ESU 6 |  |
|  | Manaus, Amazonas, Brazil | KM489413 | ESU 6 |  |
|  | Manaus, Amazonas, Brazil | KM489412 | ESU 6 |  |
|  | Cuiaba, Mato Grosso, Brazil | KM489411 | ESU 6 |  |
|  | Cuiaba, Mato Grosso, Brazil | KM489410 | ESU 6 |  |
|  | Eusebio, Ceara, Brazil | KM489409 | ESU 6 |  |
|  | Peter Parish, Saint, Dominica | KM489417 | ESU 6 |  |
|  | Peter Parish, Saint, Dominica | KM489416 | ESU 6 |  |
|  | Belo Horizonte, Minas Gerais, Brazil | KM489429 | ESU 6 |  |
|  | Belo Horizonte, Minas Gerais, Brazil | KM489428 | ESU 6 |  |
|  | Belo Horizonte, Minas Gerais, Brazil | KM489427 | ESU 6 |  |
|  | Belo Horizonte, Minas Gerais, Brazil | KM489426 | ESU 6 |  |
|  | Sao Jose, Santa Catarina, Brazil | KM489425 | ESU 6 |  |
|  | Sao Jose, Santa Catarina, Brazil | KM489424 | ESU 6 |  |
|  | Sao Jose, Santa Catarina, Brazil | KM489423 | ESU 6 |  |
|  | Brazil | HQ660055 | ESU 6 |  |
| ***Sarasinula plebeia*** | |  |  |  |
|  | Florida, USA | KM489498 | ESU 1 |  |
|  | Hawaii, USA | KM489499 | ESU 1 |  |
|  | El Hatillo, Venezuela | KM489393 | ESU 1 |  |
|  | Colombia, Caldas Laranjal, Colombia | KM489399 | ESU 1 |  |
|  | Tongonape, Lambayeque, Peru | KM489398 | ESU 1 |  |
|  | Tongonape, Lambayeque, Peru | KM489397 | ESU 1 |  |
|  | Tongonape, Lambayeque, Peru | KM489396 | ESU 1 |  |
|  | Sao Paulo, Sao Paulo, Brazil | KM489395 | ESU 1 |  |
|  | Sao Paulo, Sao Paulo, Brazil | KM489394 | ESU 1 |  |
|  | El Hatillo, Venezuela | KM489392 | ESU 1 |  |
|  | El Hatillo, Venezuela | KM489391 | ESU 1 |  |
|  | El Hatillo, Venezuela | KM489390 | ESU 1 |  |
|  | El Hatillo, Venezuela | KM489389 | ESU 1 |  |
|  | Caracas, Venezuela | KM489388 | ESU 1 |  |
|  | Caracas, Venezuela | KM489387 | ESU 1 |  |
|  | Caracas, Venezuela | KM489386 | ESU 1 |  |
|  | Caracas, Venezuela | KM489385 | ESU 1 |  |
|  | Honduras | KM489384 | ESU 1 |  |
|  | Honduras | KM489383 | ESU 1 |  |
|  | Puerto Rico | KM489382 | ESU 1 |  |
|  | Safune District, Savai'i, Samoa | KM489381 | ESU 1 |  |
|  | Philippines | KM489380 | ESU 1 |  |
|  | St. George East, Trinidad and Tobago | KM489379 | ESU 1 |  |
|  | Viamao, Rio Grande do Sul, Brazil | KM489378 | ESU 1 |  |
|  | Mexico | KM489377 | ESU 1 |  |
|  | Captain Bruce, Marigot, Saint Andrew, Dominica | KM489376 | ESU 1 |  |
|  | Miami, Florida, USA | KM489375 | ESU 1 |  |
|  | Wellington, Florida, USA | KM489374 | ESU 1 |  |
|  | Dominican Republic | KM489373 | ESU 1 |  |
|  | Mount Tapachau, Northern Mariana Islands | KM489372 | ESU 1 |  |
|  | Guatemala | KM489371 | ESU 1 |  |
|  | Panama | KM489370 | ESU 1 |  |
|  | Hawaii, USA | KM489368 | ESU 1 |  |
|  | Viet Nam | KM489367 | ESU 1 |  |
|  | Tahiti, France | KM489369 | ESU 1 |  |
|  | Iquitos, Loreto, Peru | KM489401 | ESU 6 |  |
|  | Iquitos, Loreto, Peru | KM489400 | ESU 6 |  |
|  | Rio Grande do Sul, Brazil | JX532107 | ESU 1 |  |
|  | Batanes, Batan Island, Philippines | JQ582279 | ESU 31 |  |
|  | Batanes, Batan Island, Philippines | JQ582278 | ESU 31 |  |
|  | Batanes, Batan Island, Philippines | JQ582277 | ESU 31 |  |
| ***Salasinula* sp. 1** | |  |  |  |
|  | Chapeco, Santa Catarina, Brazil | KM489501 | ESU 3 |  |
|  | Chapeco, Santa Catarina, Brazil | KM489502 | ESU 3 |  |
|  | Sao Paulo, Sao Paulo, Brazil | KM489446 | ESU 3 |  |
|  | Pinhalzinho, Sao Paulo, Brazil | KM489448 | ESU 4 |  |
|  | Chapeco, Santa Catarina, Brazil | KM489442 | ESU 3 |  |
|  | Chapeco, Santa Catarina, Brazil | KM489441 | ESU 3 |  |
|  | Chapeco, Santa Catarina, Brazil | KM489440 | ESU 3 |  |
|  | Chapeco, Santa Catarina, Brazil | KM489439 | ESU 3 |  |
|  | Chapeco, Santa Catarina, Brazil | KM489438 | ESU 3 |  |
|  | Chapeco, Santa Catarina, Brazil | KM489437 | ESU 3 |  |
|  | Sao Paulo, Sao Paulo, Brazil | KM489445 | ESU 3 |  |
|  | Caieiras, Sao Paulo, Brazil | KM489444 | ESU 3 |  |
|  | Sao Paulo, Sao Paulo, Brazil | KM489443 | ESU 3 |  |
|  | Pinhalzinho, Sao Paulo, Brazil | KM489447 | ESU 4 |  |
| ***Salasinula* sp. 2** | |  |  |  |
|  | Jatai, Goias, Brazil | KM489452 | ESU 7 |  |
|  | Jatai, Goias, Brazil | KM489454 | ESU 7 |  |
|  | Jatai, Goias, Brazil | KM489456 | ESU 7 |  |
|  | Jatai, Goias, Brazil | KM489455 | ESU 7 |  |
|  | Jatai, Goias, Brazil | KM489453 | ESU 7 |  |
|  | Jatai, Goias, Brazil | KM489451 | ESU 7 |  |
|  | Jatai, Goias, Brazil | KM489450 | ESU 7 |  |
|  | Jatai, Goias, Brazil | KM489449 | ESU 7 |  |
| ***Salasinula* sp.** | |  |  |  |
|  | Dominica | KM489500 | ESU 1 |  |
| ***Semperula wallacei*** | |  |  |  |
|  | Sabah, Malaysia | DQ897673 | ESU 28 |  |
|  | Tutuila, American Samoa | JX532109 | ESU 29 |  |
|  | Miyako Island, Okinawa, Japan | LC415572 | ESU 28 | TUMo-HC6220 |
|  | Irabu Island, Okinawa, Japan | LC415573 | ESU 28 | TUMo-HC6221 |
|  | Naha, Okinawa Island, Okinawa, Japan | LC415574 | ESU 28 | TUMo-HC7455 |
|  | Chichijima Island, Ogasawara, Tokyo, Japan | LC415571 | ESU 28 | TUMo-HC7454 |
| ***Vaginulus taunaisii*** | |  |  |  |
|  | Brazil | HQ660056 | ESU 19 |  |
| ***Veronicella cubensis*** | |  |  |  |
|  | Hawaii, USA | HQ660057 | ESU 22 |  |
|  | St. Paul Parish, Antigua and Barbuda | JX532112 | ESU 23 |  |
|  | Winfried Gibbons Nature Reserve, Devonshire Parish, South Road, Bermuda | KC206184 | ESU 22 |  |
|  | O'ahu, Hawaii, USA | HIDNA023-14 | ESU 22 |  |
| **Veronicellidae sp. *sense* Hirano et al. (2019b)** | |  |  |  |
|  | Nagura, Ishigaki Island, Okinawa, Japan | LC415569 | ESU 30 | TUMo-HC7456 |
| **Veronicellidae sp.3** | |  |  |  |
|  | El Torno, Santa Cruz, Bolivia | KM489481 | ESU 17 |  |
|  | Savedro, Santa Cruz, Bolivia | KM489484 | ESU 17 |  |
|  | Savedro, Santa Cruz, Bolivia | KM489483 | ESU 17 |  |
|  | Savedro, Santa Cruz, Bolivia | KM489482 | ESU 17 |  |
|  | El Torno, Santa Cruz, Bolivia | KM489480 | ESU 17 |  |
| **Veronicellidae sp.** | |  |  |  |
|  | SE of town, hill top shrine, Bokpyin, Tanintharyi, Myanmar | MF983692 | ESU 27 |  |
| **Outgroups** | |  |  |  |
| ***Onchidella floridana*** | |  |  |  |
|  |  | HQ660035 |  |  |
| ***Onchidium vaigiense*** | |  |  |  |
|  |  | HQ660040 |  |  |

HIDNA023-14 is deposited in BOLD. Other sequences are deposited in GenBank.
