# Supplementary material for: Species identification of introduced veronicellid slugs in Japan
Source: PeerJ. 2022 Apr 22;10:e13197. doi: 10.7717/peerj.13197 (PMC9037128; doi:10.7717/peerj.13197)
Supplement: Supplemental Information 3 [file peerj-10-13197-s003.docx]

**Table S2.** Variation of number of the tubes of the digitiform gland in each ESU.

| Specimen ID | Number of the tubes of the digitiform gland |
| --- | --- |
|  |  |
| **ESU 1 (*Sarasinula plebeia*)** |  |
| TUMo-95 | 7 |
| TUMo-96 | 7 |
| TUMo-kin7 | 8 |
| TUMo-2020o48 | 4 |
| **ESU 28 (*Semperula wallacei*)** |  |
| TUMo-kin8 | 13 |
| TUMo-HC6219 | 14 |
| TUMo-HC6220 | 13 |
| TUMo-HC6221 | 12 |
| TUMo-HC7454 | 15 |
| TUMo-HC7455 | 16 |
| **ESU 29 (*Semperula wallacei*)** |  |
| TUMo-2020o15 | 16 |
| TUMo-HC12299 | 11 |
| **ESU 30 (Veronicellidae sp.)** |  |
| TUMo-kin3 | 9 |
| TUMo-kin4 | 9 |
| TUMo-kin22 | 8 |
| TUMo-HC7456 | 5 |
| **ESU 31 (*Laevicaulis alte*)** |  |
| TUMo-2020o19 | 17 |
| TUMo-2020o48 | 14 |
| TUMo-kin11 | 10 |
| TUMo-kin13 | 11 |
| TUMo-kin15 | 15 |
| TUMo-kin16 | 14 |
| TUMo-kin17 | 14 |
| TUMo-kin20 | 13 |
| TUMo-kin24 | 18 |
